# Transmission Network of Measles During the Yamagata Outbreak in Japan, 2017

**DOI:** 10.2188/jea.JE20200455

**Published:** 2022-02-05

**Authors:** Tetsuro Kobayashi, Hiroshi Nishiura

**Affiliations:** 1 Kyoto University School of Public Health, Kyoto, Japan; 2 CREST, Japan Science and Technology Agency, Saitama, Japan; 3 Graduate School of Medicine, Hokkaido University, Sapporo, Japan

**Keywords:** epidemic, transmissibility, Paramyxoviridae, mathematical model, statistical estimation

## Abstract

**Background:**

A measles outbreak involving 60 cases occurred in Yamagata, Japan in 2017. Using two different mathematical models for different datasets, we aimed to estimate measles transmissibility over time and explore any heterogeneous transmission patterns.

**Methods:**

The first model relied on the temporal distribution for date of illness onset for cases, and a generation-dependent model was applied to the data. Another model focused on the transmission network. Using the illness-onset date along with the serial interval and geographical location of exposure, we reconstructed a transmission network with 19 unknown links. We then compared the number of secondary transmissions with and without clinical symptoms or laboratory findings.

**Results:**

Using a generation-dependent model (assuming three generations other than the index case), the reproduction number (*R*) over generations 0, 1, and 2 were 25.3, 1.3, and <0.1, respectively, explicitly yielding the transmissibility over each generation. The network data enabled us to demonstrate that both the mean and the variance for the number of secondary transmissions per primary case declined over time. Comparing primary cases with and without secondary transmission, high viral shedding was the only significant determinant (*P* < 0.01).

**Conclusions:**

The *R* declined abruptly over subsequent generations. Use of network data revealed the distribution of the number of secondary transmissions per primary case and also allowed us to identify possible secondary transmission risk factors. High viral shedding from the throat mucosa was identified as a potential predictor of secondary transmission.

## INTRODUCTION

Measles, a highly infectious disease, is caused by a virus belonging to the *Morbillivirus* genus in the Paramyxoviridae family.^1^ Typical symptoms and clinical signs of measles include fever, maculopapular rash, and catarrhal manifestations (such as conjunctivitis, coryza/runny nose, pharyngeal pain, and cough), usually following an incubation period of 10 days (range, 8 to 23 days) after exposure.^2^^–^^7^ Owing to its substantial capacity for airborne transmission, the virus can be transmitted in an open space, yielding a large basic reproduction number; the average number of secondary cases generated by a single primary case in a fully susceptible population has been documented to range from 10 to 20.^8^^–^^11^ Immunization with the measles-containing vaccine (MCV) is an effective and promising preventive option, but the disease has yet to be eliminated from many parts of the world. The main difficulties in achieving disease control include high viral transmissibility, vaccination failure (both primary and secondary failure), heterogeneous transmission patterns over geographic space (eg, persistent transmission in underdeveloped countries and exportation to highly vaccinated industrialized countries), and insufficient vaccination coverage, making it difficult to anticipate when this virus might be eventually eliminated.^3^^,^^7^^,^^12^^–^^26^

MCV was initially introduced in the mid-1960s in Japan with public subsidy. Vaccination against measles has been accepted as part of the routine immunization program since 1978. With this effort, the persistent chains of transmission for the local D5 strain were interrupted, with the last isolation of this genotype in May 2010.^4^^,^^27^^,^^28^ Japan was verified as measles-free in 2015. Nevertheless, importations coupled with clustered susceptible individuals can easily allow outbreaks to occur, sometimes accompanied by multiple transmission chains. Moreover, the scientific validity of the definition of elimination has been debated.^29^^–^^32^ Epidemics outside of Japan and multiple introductions of imported cases have led to multiple cases of local transmission, as highlighted in recently published studies.^4^^,^^27^^,^^28^^,^^33^^–^^36^

In the present study, we analyzed a measles outbreak that occurred in Japan in 2017. The outbreak began with a clustering of cases at a driving school in Yamagata Prefecture, in northeastern Japan. The datasets offered us the opportunity to reconstruct a transmission network, which we used to determine who acquires infection from whom (WAIFW). Whereas this type of reconstruction has been reported in previous studies for a variety of diseases (eg, plague, influenza, Middle East respiratory syndrome, and measles^35^^,^^37^^–^^40^), use of such an analysis for evaluating control programs has yet to be established. Whereas some studies show the serial interval, which is defined as the time interval from illness onset in a primary case to illness onset in a secondary case, and have also estimated the effective reproduction number over time,^37^^,^^38^^,^^40^^,^^41^ the differential usefulness of the temporal distribution alone and the network data in a public health context has yet to be discussed. Compared with the epidemic curve (or temporal distribution of cases) alone, we might ask what the transmission network can offer in terms of additional insights into the control of an infectious disease.

In the present study, we aimed to estimate measles transmissibility over time and explore any heterogeneous transmission patterns, clarifying different aspects of the information that can be extracted from analyzing the epidemic curve and the transmission network. For the purpose of exposition, we chose to analyze the abovementioned 2017 measles outbreak in Yamagata, Japan, which included 60 confirmed cases of measles. As the local government of Yamagata performed contact tracing and raised public awareness via swift media announcements, we evaluated the control measures by estimating the generation-dependent reproduction number (*R*), which we defined as the average number of secondary cases produced by a single primary case in each respective generation, using our original generation-dependent model. Moreover, because of the practice of intensive contact tracing, the transmission network was mostly known (ie, epidemiologically traced). For a small number of unobserved parts of the network, we could partially reconstruct the transmission tree by quantifying the weight of edges for each of the unobserved potential links. By reconstructing the network of WAIFW, we explored the possible determinants for the primary cases causing secondary transmissions.

## METHODS

### Epidemiological data

Adhering to the Infectious Disease Law of Japan, physicians are mandated to notify all diagnosed measles cases to the government. Such notified cases include those confirmed by laboratory testing (ie, reverse-transcriptase polymerase chain reaction [RT-PCR]) or with elevated IgM antibody levels using paired serum, or clinically diagnosed patients who exhibit all three clinical signs or symptoms (fever, rash, and catarrhal symptoms).^42^ In addition, laboratory-confirmed modified measles cases that do not satisfy all the aforementioned triad of typical measles are also notified in the surveillance system. For each case, the so-called “line list” is publicly announced by the local municipal government. The report for each case includes the date of onset, date of diagnosis, sex, age group (10-year age groups), residential city and prefecture, and the estimated place of infection (or traced link to an earlier case).

In the present study, we used two independent information sources for the 2017 outbreak in Yamagata.^43^^,^^44^ One is the abovementioned line list announced by Yamagata City, the capital of Yamagata Prefecture in Northeast Japan.^44^ However, by relying only on Yamagata City data, the transmission tree was incomplete. Specifically, the data mostly included the documentation of links that arose from the index case only. Therefore, we also explored another data source that provided more detailed information of the transmission tree and also laboratory test results using real-time RT-PCR to quantify the viral load in throat swab samples.^43^ The presence of clinical signs and symptoms for each case were referenced from a published study.^43^ Even using both data sources, several links to the primary case were missing for 19 of the observed cases; therefore, we sought the possible primary cases for these cases and reconstructed the transmission tree in the following analysis.

### Description of the outbreak

Here, we briefly describe the outbreak. The index case was a Japanese man in the age group 20–29 years who had just returned from a short trip to Bali, Indonesia, on February 26, 2017. His symptoms started on March 3, 2017, one day following his temporary relocation to Yamagata (approximately 300 km north of Yokohama), to which he traveled by bullet train to attend a short course at a driving school to obtain his driving license. Of the 59 secondary cases, the links (or primary cases) for 19 remained unknown. However, among the other secondary cases, 25 were traced and belonged to the first generation (ie, they were directly infected by the index case or were generation zero) whereas 15 were second-generation cases (ie, infected by the first generation). All 25 first-generation cases were exposed either at the driving school or at a hotel where the index case and driving school participants stayed and shared the same indoor air space.

**Figure 1.**
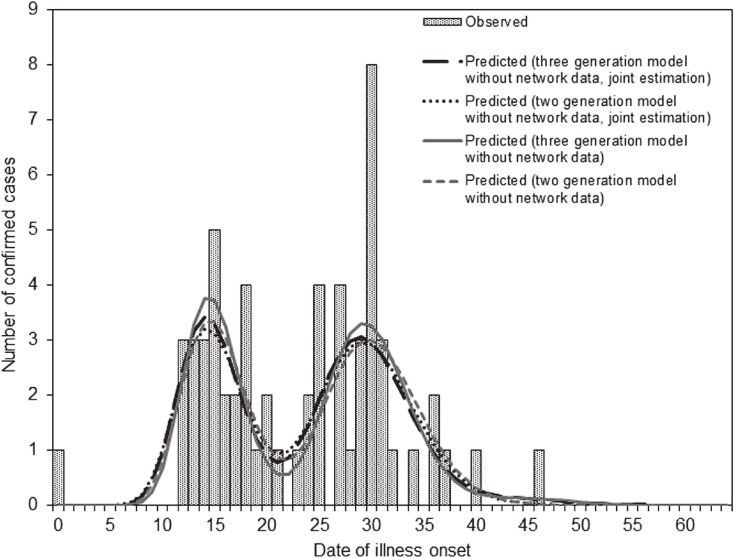
Temporal distribution of measles cases in Yamagata, Japan in 2017. Bars represent confirmed cases as a function of the date of illness onset; continuous lines represent the predicted number of cases. A temporal model (Model 1) was used; assuming there were two and three generations excluding the index case, differing results are shown. For an assumed number of generations, a joint estimation of the generation-dependent reproduction numbers and the parameters governing the serial interval distribution was considered whereas the other model estimated only the generation-dependent reproduction numbers and fixed parameters for the serial interval. Different lines are used for two- and three-generation models with and without use of network data for estimation of the serial interval distribution.

**Figure 2.**
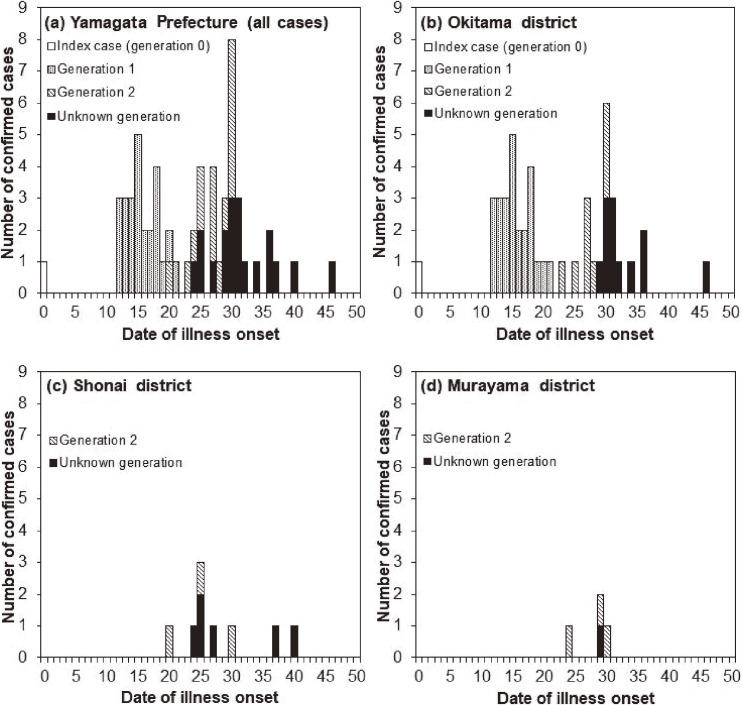
Epidemic curves of 2017 measles outbreak in Yamagata, Japan, by geographic region. A. Epidemic curve for all of Yamagata Prefecture. B. Cases diagnosed and notified in Okitama, C. Shonai and Murayama districts. In all panels, bars are shown in different patterns according to generation.

The temporal distribution of measles cases in Yamagata Prefecture is shown in Figure 1. The epidemic curves in three discrete regions (Okitama, Shonai, and Murayama districts) of Yamagata Prefecture are shown in Figure 2 (in which the bar patterns represent the generations); 46, 9, and 4 cases occurred in these districts, respectively, except for the index case.

### Model 1: Temporal model

We used two different modeling approaches to the two different types of datasets, namely, temporal distribution-only data and transmission-network data, to clarify the practical importance of the transmission network data. In the first model, we performed an analysis of the temporal distribution of the cases alone. Subsequently, using the second model, we partially reconstructed the network, to show what information could be additionally extracted compared with the temporal data alone.

Before conducting analysis using the temporal model, we first estimated the serial interval distribution. For this purpose, using the line list with known illness onset dates, we allocated a unique case number, *i*, to each of the 60 cases according to the order of the reporting date, with *i* = 1 being the index case. We divided the 60 cases into two groups: (i) cases whose links to the primary case were known, denoted by *W*, and (ii) cases without known links to the primary case, denoted by *U*. Letting the date of illness onset in the index case be *t*_0_ = 0, the time of illness onset in case *i*, *t*_i_ such that *i* ∈ [2,60] can be interpreted as the number of days since illness onset of the index case. The serial interval between the primary case *v*(*i*) and the secondary case *i* is expressed as *t*_i_ − *t*_v(i)_. The serial interval distribution for other infectious diseases (eg, plague and foot and mouth disease^40^^,^^45^) have been empirically fitted to the gamma distribution; for example, Klinkenberg and Nishiura have used the gamma distribution for the generation time of measles.^46^ Similarly, the serial distribution *g*(.) of case *i* (*i* > 1) in the Yamagata outbreak was assumed to be governed by the following gamma distribution:

$$g(t_{i}-t_{v(i)};k,\theta )=(t_{i}-t_{v(i)}{)}^{k-1}\frac{\exp(-(t_{i}-t_{v(i)})/\theta )}{\Gamma (k)\theta^{k}}$$ (1)

where *k* and *θ* are the shape and scale parameters, respectively.

Next, we estimated the generation-dependent reproduction number from the temporal distribution. Specifically, we estimated *R*_n_, which represents the average number of secondary cases generated by a single primary case in generation *n*. This type of generation-dependent formulation has been used elsewhere,^41^^,^^47^ and we discuss the derivation process in eMaterials 1. Together with the parameters used for the serial interval distribution, the total likelihood function for estimating the reproduction numbers is:

$$L(\Theta ;t_{i})=\prod_{i\in W}g(t_{i}-t_{v(i)})\prod_{i\in [1,60]}\frac{I(t_{i})}{\int_{0}^{\infty }I(s)ds}.$$ (2)

It should be noted that the function *I*(*t*_i_) also contains two parameters (*k* and *θ*) that were originally required for describing *g*(*t*_i_ − *t*_v(i)_). It should also be noted that the normalization constant for the incidence function is equal to the cumulative number of cases, excluding the index case (ie, equal to 59). In addition to the likelihood (2), we also considered circumstances where there is no access to the serial interval data. In such circumstances, estimation of parameter *Θ* relies on the epidemic curve alone. That is, estimation using (2) is based on the epidemic curve and the transmission network data whereas the use of likelihood without network data for *g*(.) relies on the epidemic curve only. Subsequent to the estimation, we computed the epidemic curve described by *I*(*t*_i_) and visually compared the solution against the observed epidemic curve.

### Model 2: Network-based model

Here, we partially reconstructed the transmission tree. To do so, we took into account both the serial intervals and the Euclid distance between the residence of the potential primary case and that of the secondary case, as measured in kilometers. Let *x*_iv(i)_ be the measured Euclidian distance between *i* and *v*(*i*). Assuming that the dependence mechanism of secondary transmission on the geographic distance is sufficiently captured by an exponential decay model with a decay rate of distance, *λ*, the likelihood function to estimate the serial interval distribution and *λ* is then written as

$$\begin{array}{rl} & L(k,\theta ,\lambda |t_{i}-t_{\text{v}(i)},x_{i\text{v}(i)})\\ & \;=\prod_{j\neq i}\prod_{i\in W}\frac{g(t_{i}-t_{\text{v}(i)}|k,\theta )\exp(-\lambda x_{i\text{v}(i)})}{\sum g(t_{i}-t_{j}|k,\theta )\exp(-\lambda x_{ij})} \end{array}$$ (3)

Using the estimated values of *k*, *θ*, and *λ*, we calculated the probability, *P_j_*(*i*), that a secondary case *j* {*j* ∈ *U*} is linked to a potential primary case *i*, such that:

$$P_{j}(i)=\frac{g(t_{j}-t_{i}|k,\theta )\exp(-\lambda x_{ji})}{\sum_{i\neq j}g(t_{j}-t_{i}|k,\theta )\exp(-\lambda x_{ji})}$$ (4)

To deterministically reconstruct the transmission network, we took the highest probability algorithm, selecting the link that yields the highest value of *P_j_*(*i*) among all possible candidates for *j*.

Subsequently, we used the tree to directly calculate the number of secondary transmissions generated by each of the 60 cases during the outbreak, visually counting the reproduction number of each generation.

### Exploring the risk factors for secondary transmission

Using the reconstructed transmission tree, symptom data, and viral shedding status (see eTable 1) of each case, we explored the possible epidemiological variables that could explain the number of secondary transmissions per single primary case. To examine the potential association between a symptom and secondary transmission, we divided the 60 cases into two groups, specifically, cases with or without a particular symptom. For each symptom and for each laboratory testing result that could potentially explain transmissibility, we performed Wilcoxon’s rank-sum test to compare the number of secondary transmissions per primary case between the two groups. We used the non-parametric test here, because the distribution of the number of secondary cases per single primary case was highly skewed. When the link for secondary transmission was not deterministically reconstructed and there were two equally plausible primary case candidates, we allocated 0.5 as the weight for each candidate edge, for comparative purposes.

### Ethical considerations

The present study examined publicly available data that lacked any personally identifiable information. As such, ethical approval was not required for the present study.

### Data sharing statement

The case series data used in this study are available as online supporting material (https://www.jstage.jst.go.jp/browse/jea/).

## RESULTS

When model 1 (ie, the temporal model) was used, the epidemic curve was modeled to involve two main humps, one reflecting the first generation and the other the second generation (Figure 1). The curve drawn in the three-generation model (in which case *R* of the second generation, *R*_2_, takes a non-zero value) shows a very small peak as the third generation at around day 42 to day 50. The three-generation model yielded estimates of the reproduction numbers of generations 0, 1 and 2, each denoted by *R*_0_, *R*_1_, and *R*_2_, respectively, at 25.3 (the 95% confidence interval [CI] was not calculable), 1.3 (95% CI, 0.7–2.2), and <0.1 (95% CI, 0.0–0.2) (Table 1), which implied that the outbreak was brought under control by the second generation. The mean and variance of the serial interval distribution were estimated to be 14.8 days (95% CI, 14.2–15.4) and 9.1 days^2^ (95% CI, 6.7–12.9), respectively. Estimated mean and variance in the other models are summarized in Table 1. When the two-generation model was used, *R*_0_ and *R*_1_ were estimated at 25.5 (95% CI not calculable) and 1.3 (95% CI, 0.8–2.3), respectively; again, the outbreak was ongoing at the first generation and subsequently brought under control by the second generation (because *R*_2_ was assumed to be zero by definition). Applying model 1 (ie, temporal model) with two and three assumed generations (except for the index-case generation) and using network data, the Akaike information criteria (AIC) for the three-generation model with four parameters (AIC = 611.3) was slightly lower than that of the two-generation model with three parameters (AIC = 611.7), and this finding was independent of the use of network data for estimating the serial interval (Table 1). No improvement in the AIC was observed by assuming four or more generations (results not shown). When the serial interval distribution was quantified from the epidemic curve alone (ie, when we did not jointly use the transmission network data), the resulting estimate of *R*_n_ for all *n* did not significantly deviate from that using both the epidemic curve and transmission network data for all generations *n* (for *n* ≥ 0) (Table 1).

**Table 1.** Parameter estimates of model 1 with assumed two and three generations (except for index case), each with simultaneous serial interval estimations

Model description	*R*_0_	*R*_1_ (95% CI)	*R*_2_ (95% CI)	*k* (95% CI)	*θ* (95% CI)	Mean SI (95% CI) days	Variance of SI (95% CI) days^2^	Model parameters	AIC
Three-generation model using network data	25.3	1.3 (0.7, 2.2)	<0.1 (0.0, 0.2)	24.1 (17.2, 32.2)	0.6 (0.5, 0.9)	14.8 (14.2, 15.4)	9.1 (6.7, 12.9)	4	611.3

Two-generation model using network data	25.5	1.3 (0.8, 2.3)	N/A	21.5 (15.6, 28.5)	0.7 (0.5, 1.0)	14.9 (14.3, 15.6)	10.4 (7.8, 14.4)	3	611.7

Three-generation model without using network data	25.7	1.2 (0.7, 2.1)	<0.1 (0.0, 0.2)	29.8 (18.5, 43.8)	0.5 (0.3, 0.8)	14.9 (14.2, 15.7)	7.5 (5.0, 12.3)	4	403.3

Two-generation model without using network data	26.0	1.3 (0.7, 2.2)	N/A	23.5 (15.0, 34.2)	0.6 (0.4, 1.0)	15.2 (14.4, 16.0)	9.8 (6.7, 15.6)	3	404.4

*R*_n_ represents the average number of secondary cases generated by a single primary case in generation *n*. *k*, scale parameter and *θ*, shape parameter of the serial interval.

It should be noted that the dataset of transmission network was used for the two models “using network data” whereas this was not the case for the two models “without use of network data”; therefore the AIC cannot be compared among them. Abbreviations: SI: serial interval; AIC: Akaike information criteria; CI: confidence interval; R^2^, coefficient of determination.

**Figure 3.**
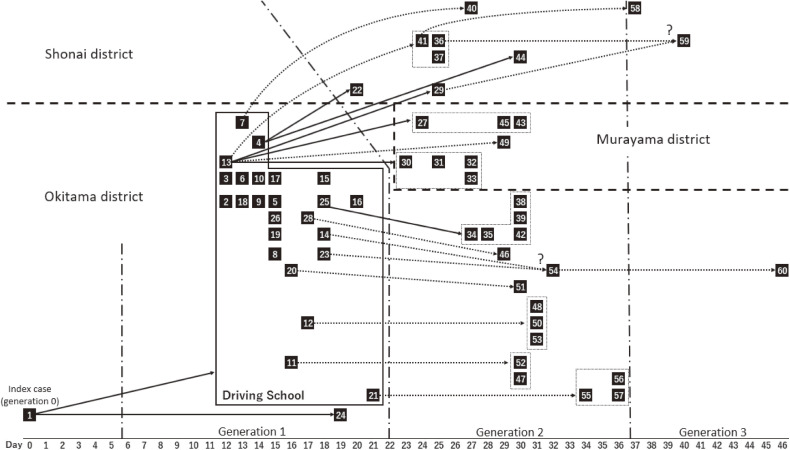
Reconstructed transmission tree for the 2017 measles outbreak in Yamagata, Japan. Each black square represents an individual confirmed measles case who was allocated a unique number from 1 (index) to 60, according to the notification order (model 2). The transmission network, based on the observed contact data (eg, sharing a confined space or verified family contact), is shown by solid arrows. Reconstructed transmission network based on our inference, using the time-lag of illness-onset times and geographic area of residence, as shown by the dotted arrows. When a single primary case was not identified, two or more dotted arrows were drawn for all possible networks, followed by a question mark. The driving school acted as the focal area for transmission in the beginning of the epidemic; the traced contacts within the driving school are grouped using large black squares. Variable-sized dotted squares represent the grouping of a cluster of cases that was generated from an identical primary case.

The transmission network was then reconstructed in another analysis using model 2 (Figure 3). It is remarkable that we were able to calculate not only the number of secondary cases per individual, but that we could trace the secondary transmissions by the most plausible place of transmission (eg, the driving school), by geographic district, and also by generation. As for the geographic distance, the likelihood equation (3) yielded the maximum likelihood estimate of decay rate, *λ*, at 0.02 (95% CI, 0.00–0.03) per kilometer. Deterministically reconstructing the transmission network among 19 secondary cases without known primary cases, by choosing for each secondary case a single link with the highest probability out of all possible links, we identified the “likeliest” primary cases for 17 secondary cases. Only two cases (ie, cases 54 and 59), in whom the illness developed on day 32 and day 40, respectively, were not linked to a single unique case (and had two or more possible primary cases). Within the network, it was considered that the outbreak was terminated at generation three. In addition to the primary case who generated an extraordinarily large number of secondary cases, there were two superspreaders: (i) the index case who contributed to transmissions at the driving school, and case 13 who acquired the infection from the index case and became ill on day 12. The AIC value of model 2 (ie, network-based model) was 442.5 (with three parameters, ie, two for serial interval distribution and one for decay rate by distance); this was greater than the AIC values of model 1 that used two- and three-generation models and network data, which were 403.3 (with three parameters) and 404.4 (with four parameters), respectively.

**Figure 4.**
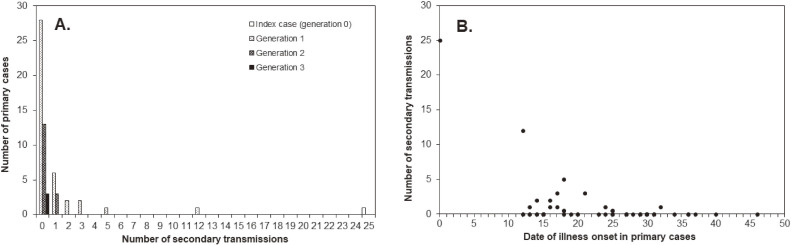
Generation- and time-dependent variations in the number of secondary measles transmissions (all derived from model 2). A. Offspring distributions for the number of secondary transmissions per single primary case are shown by generation. The vertical axis shows the number of primary cases measuring the frequency; the horizontal axis shows the number of secondary transmissions per single primary case. B. Time-dependent variations in the number of secondary transmissions per single primary case. The number of secondary transmissions is shown as a function of the date of illness onset in each primary case.

From the reconstructed transmission tree in model 2, we extracted the distribution of the number of secondary transmissions per primary case (Figure 4A). Overall, the mean number of secondary transmissions per primary case, irrespective of generation, was 0.98, which is comprehensible from the analysis of the entire epidemic curve (ie, the mean should be (*n* − 1)/*n* where *n* is the cumulative count of cases, amounting to 60 in the Yamagata outbreak), with a variance of 12.8. For generations 0, 1, 2, and 3, the estimated mean number of secondary transmissions was 25.0, 0.8, 0.2, and 0.0 cases (per primary case), respectively. Similarly, the variance for generations 0, 1, 2, and 3 was estimated at 576.8, 4.4, 0.8, and 1.0 cases^2^, respectively. Figure 4B shows the number of secondary transmissions per primary case as a function of the date of illness onset for the primary case, indicating that both the mean value and variance declined over time.

Table 2 summarizes the comparison of the number of secondary transmissions between primary cases with and without particular symptoms or laboratory testing characteristics. Cases with skin rash (*P* < 0.01) and high viral shedding (*P* < 0.01) were more likely to have a greater number of secondary cases associated with them than cases lacking these characteristics. Clinical forms of measles being typical or atypical were not associated with the number of secondary transmissions (*P* = 0.49). Conditioning the comparison among the primary cases who had at least one suspected secondary transmission, high viral shedding was the only significant determinant associated with heterogeneous patterns of the secondary transmissions (*P* < 0.01). In both series of comparisons, cough, runny nose, sore throat, and headache did not show any significant association with the number of secondary transmissions.

**Table 2.** Comparison of the number of secondary infections per person between those with and without each symptom

	Among all cases (absence vs presence of characteristics)			Among cases with at least one secondary case (absence vs presence of characteristics)		

Clinical characteristics	Median [interquartile range]	Mean	*P*-value (Wilcoxon)	Median [interquartile range]	Mean	*P*-value (conditioned)
Rash	0.00 [0.00, 0.00] vs 0.50 [0.00, 2.00]	0.23 vs 2.66	<0.01^a^	1.00 [0.75, 2.50] vs 1.50 [0.50, 6.75]	1.50 vs 5.05	0.71
Stomatitis	0.00 [0.00, 0.50] vs 0.00 [0.00, 0.00]	1.14 vs 0.00	0.56	NC	NC	NC
Cough	0.00 [0.00, 0.50] vs 0.00 [0.00, 0.50]	1.01 vs 1.79	1.00	1.00 [0.75, 3.00] vs 6.30 [0.50, 12.00]	3.50 vs 6.25	1.00
Runny nose	0.00 [0.00, 0.63] vs 0.00 [0.00, 0.50]	1.24 vs 0.17	0.90	2.00 [1.00, 4.00] vs 0.50 [0.50, 0.50]	4.38 vs 0.50	0.07
Sore throat	0.00 [0.00, 0.50] vs 0.00 [0.00, 1.00]	1.19 vs 0.40	0.67	2.00 [0.50, 4.00] vs 1.00 [1.00, 1.00]	4.31 vs 1.00	0.66
Conjunctivitis	0.00 [0.00, 0.50] vs 0.00 [0.00, 0.00]	1.18 vs 0.00	0.28	NC	NC	NC
Headache	0.00 [0.00, 0.50] vs 0.00 [0.00, 0.63]	1.23 vs 0.25	1.00	2.00 [0.75, 4.00] vs 0.75 [0.50, 1.00]	4.35 vs 0.75	0.26
Arthralgia	0.00 [0.00, 0.50] vs 0.00 [0.00, 0.00]	1.16 vs 0.00	0.39	NC	NC	NC
Rash and cough	0.00 [0.00, 0.50] vs 0.50 [0.00, 0.12]	0.93 vs 4.17	0.15	1.00 [0.50, 3.00] vs 6.25 [0.50, 12.00]	3.50 vs 6.25	1.00
High viral shedding (threshold)	0.00 [0.00, 0.00] vs 8.50 [0.00, 21.8]	0.30 vs 10.50	<0.01^a^	1.00 [0.50, 2.00] vs 12.00 [5.00, 25.00]	1.31 vs 14.00	<0.01^a^
Typical measles	0.00 [0.00, 0.00] vs 0.00 [0.00, 0.13]	0.25 vs 2.25	0.05^a^	1.00 [1.00, 2.00] vs 2.00 [0.50, 8.50]	1.36 vs 5.50	0.55

NC, not calculable.

^a^Significant results. Right-hand half of the table shows the same comparison among those who had at least one or more secondary infections. Typical measles is defined by fever, rash, and catarrhal symptoms (with one or more of the following symptoms: conjunctivitis, coryza, pharyngeal pain, and cough).

## DISCUSSION

In the present study, we explored the 2017 measles outbreak in Yamagata, Japan, using two distinct methods for analyzing two different sets of data. The first analysis relied on the temporal distribution of the illness-onset dates of cases, enabling us to capture the generation structure of the epidemic and showing that the reproduction number of generation 2 was below the value of 1. This allows us to objectively state that the outbreak was brought under control by the second generation. The reproduction number of generation 0 was on the order of 25. The estimated number of secondary cases per primary case was comparable to the conventional value of the basic reproduction number, *R* = 10–20 in a fully susceptible population.^8^^–^^11^ However, the first-dose vaccination coverage in Yamagata is greater than 98% (*v* = 0.98),^48^ which should have led the effective reproduction number to take the value of (1 − *v*)*R* = 0.2–0.4 in a randomly mixing population. Thus, the estimated reproduction number of 25 in generation 0 in our study indicated that the outbreak started with a superspreading event. Comparing two- and three-generation models (model 1), the latter yielded a slightly lower AIC value, implying that there were three generations (plus the index case) in total. However, the difference of AIC values was as small as <1.0; thus, it is difficult to conclude that the three-generation model was a better fitted model.

The second part of the analysis focused on the transmission network. Using the illness onset date along with the serial interval, and also analyzing the geographic location for an exposure, a transmission network with 19 unknown links was reconstructed.^37^^,^^45^^,^^49^ Comparing AIC values between models 1 and 2 for the incidence dataset, model 1 yielded a smaller AIC value than that of model 2 (403.3 vs 442.5, respectively) and was regarded as better fitted to the observed data. In fact, model 1 is simple enough to capture the temporal incidence dynamics. However, the network data in model 2 enabled us to show that the variance as well as the mean values of the secondary transmissions produced by a single primary case declined as a function of time; the confidence interval calculated in model 1 disregards the actual distribution of the number of secondary transmissions that is skewed to zero for all generations. That is, in addition to the overall success of control by verifying *R*_n_ < 1 for *n* ≥ 2, the individual-based network reconstruction demonstrates that there were no superspreaders in the second and third generations. Overall, the control effort was successful in not allowing the emergence of a primary case who generated a large number of secondary transmissions. Moreover, by comparing the number of secondary transmissions with those who had and those who did not have clinical symptoms or laboratory characteristics, we were able to identify high viral shedding from the throat mucosa as a potential predictor of secondary transmission. The network reconstruction implied that model 2 could also estimate that there were three generations of infection following the index case; however, this idea relies on our probabilistic reconstruction of the transmission network, and the validity of this finding remains highly dependent on the validity of our reconstruction algorithms.

In addition to these practical findings, it should be noted that we were able to clarify the difference in retrievable information between using temporal data alone and using network data, as an important technical take-home message. Published studies usually rely on a single type of data analysis using a single model.^37^^,^^49^ Relying on the temporal distribution of illness onset, it is plausible that the outbreak involved a total of four generations, including the index case. We generated the generation-dependent reproduction number and interpreted the epidemic dynamics over the generation; however, such interpretation relied on the mean estimate of the number of secondary transmissions. Reconstructing the transmission network, we were able to construct a stochastic network and measure the heterogeneous patterns of transmission at individual level. With the individual dataset, we explored both the mean and variance, and an even higher moment of the number of secondary transmissions per primary case. The network also allowed us to verify that generation three was most likely to be the final generation.

Furthermore, using the additional datasets of clinical symptoms and laboratory testing results at individual level, we were able to perform group comparisons to examine the statistical associations with secondary transmission. High viral shedding from the throat of the primary case appeared to be a potentially good transmission predictor, which echoes the main findings reported elsewhere.^43^^,^^50^ Nevertheless, its use as a routine tool to find potentially infectious individuals may not be practical because measuring viral load involves a several-day delay and incurs a cost. To the best of our knowledge, the present study is the first to show how different models, applied to the same outbreak, yield different aspects of results that can be used to evaluate outbreak control. That is, the reproduction number alone is retrievable from the temporal distribution of cases (model 1) for the “overall” evaluation of success in control whereas the transmission network (model 2) can additionally help us understand the success of contact tracing (by ensuring the absence of superspreaders). Although the exact mechanism of superspreading events needs to be further elucidated, the network can be used for identifying possible clinical correlates of infectiousness (eg, virus shedding in the throat).

Additional technical points regarding the temporal model (model 1) should be discussed. When the serial interval was estimated with the reproduction numbers, the mean serial interval was estimated to be about 15 days, a slightly longer estimate than the published estimate of around 12 days.^46^^,^^51^ Although the temporal model can draw a best-fit epidemic curve, the model missed the stochastic nature of transmission in its equations, and the branching process or its analogs should ideally be applied to account for the stochasticity. Despite the model’s simplicity and deterministic nature, it should be noted that the small variance-to-mean ratio of the serial interval for measles enabled us to identify the generation structure from the epidemic curve, and an abrupt decline in the reproduction number by generation was quantitatively demonstrated. As for the reconstructed transmission tree, this also permitted us to calculate the reproduction number over time, eventually converging to a value of 0. Whereas variance in the number of secondary transmissions per primary case was calculable, it should be noted that our method missed the posterior distribution of the weight on each edge (ie, variance of linkage at individual level), which could be the subject of future improvement. Four limitations in this study should be noted. First, our model did not consider unobserved cofounding factors that could have affected the transmission dynamics. For instance, the vaccination history of diagnosed cases was incomplete and was therefore ignored during the analysis. Thus, it was not feasible to explicitly account for vaccination history and vaccine effect in our model. At a minimum, using both models 1 and 2, *R*_1_ = 1.3 indicated that the outbreak could have been brought under control from the first generation, if 23.1% of susceptible individuals had received prior vaccination. For similar reasons, there could have been better clinical datasets that defined modified measles, which might be characterized by a low transmission risk as compared with classical measles.^1^^,^^33^^,^^42^^,^^52^ Second, whereas cases were closely traced during the course of the outbreak, there might have been other individuals involved in the outbreak that were not identified, including modified measles cases that did not consult a physician. Third, because the observation data were incomplete, we had to extrapolate the onset dates for some of the cases by subtracting a constant reporting delay from the reported date. Fourth, the virus genotype was not considered for this small outbreak.^53^

In summary, we analyzed a measles outbreak in Yamagata from two different angles using different types of data and modeling approaches. Whereas both models successfully indicated that the outbreak was brought under control by generation three and the reproduction number declined over the course of the outbreak, we have shown that use of network data can yield the distribution of the number of secondary transmissions; moreover, the reconstructed tree allowed for the identification of possible risk factors of secondary transmission. Depending on the study objectives and available data during an outbreak investigation, appropriate methods should be chosen to retrieve pertinent information. That is, if the overall success of interventions must be evaluated using the reproduction number, use of only the temporal distribution of cases might suffice. Nevertheless, to explore individual variations in secondary transmission (and verify that contact tracing did not allow for the emergence of superspreaders) or to attribute the transmission to individual factors (eg, clinical symptoms), we have shown that network data can serve as a very useful source of information.

## APPENDIX A. SUPPLEMENTARY DATA

eMaterials 1. Derivation of generation-dependent model

eTable 1. Epidemiological profiles of the 60 confirmed cases during the 2017 measles outbreak in Yamagata, Japan
