# Supplementary material for: Transmission Network of Measles During the Yamagata Outbreak in Japan, 2017
Source: J Epidemiol. 2022 Feb 5;32(2):96–104. doi: 10.2188/jea.JE20200455 (PMC8761560; doi:10.2188/jea.JE20200455)
Supplement: Supplementary file 1 [file je-32-096-s001.pdf]

eMaterials 1. Derivation of generation-dependent model

We estimated the generation-dependent reproduction number from the temporal distribution. Let $i_1(t)$ represent the number of cases in the first generation (where the index case is referred to as generation zero). Also, let R_i represent the average number of secondary cases generated by a single primary case in generation i. Using R_0 and the serial interval as a function of time since the illness onset of the index case, $i_1(t)$ is described as

$$i_1(t_i; k, \theta, R_0) = R_0 g(t_i) \tag{S1}$$

The next generation $i_2(t)$ is obtained by multiplying the reproduction number of the first generation R_1, the average number of second-generation cases generated by a single case in the first generation, and taking the convolution of the serial interval for two transmission processes (i.e. from the index case to the first generation and from the first to the second generation). Accordingly, we have

$$i_2(t_i; k, \theta, R_0, R_1) = R_0 R_1 \sum_{j=1}^{i-1} g(t_i - t_j) g(t_j) \tag{S2}$$

Similarly, the epidemic curve of the third generation, $i_3(t)$, is described as

$$i_3(t_i; k, \theta, R_0, R_1, R_2) = R_0 R_1 R_2 \sum_{j=1}^{i-1} \sum_{h=1}^{j-1} g(t_i - t_j - t_h) g(t_j) g(t_h) \tag{S3}$$

where R_2 is the average number of third-generation cases generated by one second-generation case.

Supposing that there are only three generations (excluding the index case) by time t since the illness onset for the index case, the epidemic curve, $I(t)$, is described by

$$I(t_i) = i_1(t_i) + i_2(t_i) + i_3(t_i) \tag{S4}$$

Because the length of time of the epidemic was under 50 days, whereas the mean serial interval in the scientific literature is about two weeks, we assumed that the total number of generations inclusive of the index case is perhaps three or four. Because the size of the epidemic was 60, R_0 was arithmetically calculated as

$$R_0 = \frac{59}{1 + R_1 + R_1 R_2} \tag{S5}$$

In the case of the three-generation model described by equation 5, the unknown parameters Θ we would like to estimate are θ, k, R_1, and R_2.

eTable 1. Epidemiological profiles of the 60 confirmed cases during the 2017 measles outbreak in Yamagata, Japan

Case Number	Age group	Sex	Source of infection	Place of infection	Onset	Number of observed transmissions	High viral shedding[b]
1	20	M	n/a	Bali	2017/03/03	25	yes
2	10	M	1	Driving School	2017/03/15	0	no
3	10	F	1	Driving School	2017/03/15	0	no
4	20	F	1	Driving School	2017/03/17	2	no
5	20	M	1	Driving School	2017/03/18	0	no
6	10	M	1	Driving School	2017/03/16	0	no
7	40	M	1	Hotel	2017/03/16	0	no
8	10	M	1	Driving School	2017/03/18	0	no
9	30	M	1	Hotel	2017/03/17	0	no
10	30	M	1	Hotel	2017/03/17	0	no
11	20	F	1	Hotel	2017/03/19	0	no
12	20	F	1	Hotel	2017/03/20	0	no
13	30	M	1	Hotel	2017/03/15	8	yes
14	30	M	1	Driving School	2017/03/21	0	no
15	20	F	1	Driving School	2017/03/21	0	no
16	20	F	1	Hotel	2017/03/23	0	no
17	10	M	1	Driving School	2017/03/18	0	no
18	10	M	1	Driving School	2017/03/16	0	no
19	10	M	1	Driving School	2017/03/18	0	no
20	20	M	1	Driving School	2017/03/19	0	no
21	40	M	1	Driving School	2017/03/24	0	no
22	30	M	4	others	2017/03/23	0	no
23	30	M	1	Hotel	2017/03/21	0	no
24	20	M	1	others	2017/03/22	0	no
25	20	M	1	Hotel	2017/03/21	5	yes
26	30	F	1	Hotel	2017/03/18	0	no

27	30	M	13	others	2017/03/27	0	no
28	30	M	1	Driving School	2017/03/20	0	no
29	20	M	13	others	2017/03/28	0	no
30	30	M	13	others	2017/03/26	0	no
31	30	F	13	others	2017/03/28	0	no
32	0	M	13	others	2017/03/30	0	yes
33	20	M	13	others	2017/03/30	0	no
34	20	M	25	others	2017/03/30	0	no
35	20	M	25	others	2017/03/31	0	no
36[a]	40	M	unknown	others	2017/03/28	0	yes
37[a]	10	M	unknown	others	2017/03/28	0	yes
38	10	M	25	others	2017/04/02	0	no
39	30	M	25	others	2017/04/02	0	no
40	20	M	unknown	others	2017/03/30	0	no
41	50	F	unknown	others	2017/03/27	0	no
42	30	M	25	others	2017/04/02	0	no
43	40	M	13	others	2017/04/02	0	no
44	50	F	4	others	2017/04/02	0	no
45	30	F	13	others	2017/04/01	0	no
46	20	F	unknown	others	2017/04/01	0	no
47	60	M	unknown	others	2017/04/02	0	no
48	30	M	unknown	others	2017/04/03	0	no
49	30	F	unknown	others	2017/04/01	0	no
50	40	M	unknown	others	2017/04/03	0	no
51	40	M	unknown	others	2017/04/02	0	no
52	20	M	unknown	others	2017/04/02	0	no
53	30	M	unknown	others	2017/04/03	0	no
54	30	M	unknown	others	2017/04/04	0	no
55	20	M	unknown	others	2017/04/06	0	no
56	30	M	unknown	others	2017/04/08	0	no
57	30	M	unknown	others	2017/04/08	0	no
58	20	F	unknown	others	2017/04/09	0	no
59	40	F	unknown	others	2017/04/12	0	no
60	40	F	unknown	others	2017/04/18	0	no

[a] Either case 36 or 37 was known as shedding the virus

[b] High virus shedding was defined as the cut-off point of throat swab's cycle threshold at 24.78 or smaller (corresponding to 6.7×10^5 measles virus RNA copies per throat swab).
